# Identifying multimorbidity risks in older adults: a cross-sectional study using the RGA screening data

**DOI:** 10.3389/fpubh.2025.1600779

**Published:** 2025-07-28

**Authors:** Jinmyoung Cho, Allison Gibson, Max Zubatsky, Wenjin Wang, Ebow Nketsiah, Angela Sanford, Marla Berg-Weger

**Affiliations:** ^1^School of Medicine, Saint Louis University, St. Louis, MO, United States; ^2^School of Social Work, Saint Louis University, St. Louis, MO, United States; ^3^College of Social Work & Criminal Justice, Florida Atlantic University, Boca Raton, FL, United States

**Keywords:** geriatric syndromes, multimorbidity, older adults, rapid geriatric assessment (RGA), risk assessment

## Abstract

Approximately one in two older adults in the U.S. experiences multimorbidity, defined as the coexistence of two or more chronic diseases. The consequences of multimorbidity are significant, including increased vulnerability to acute illness, exacerbation of existing conditions, frequent hospitalizations, and elevated medical costs. Identifying the risk factors for multimorbidity in advance can guide healthcare service decision-making and help prevent adverse outcomes. This study examines the presence of multimorbidity (i.e., self-reported as having five or more illnesses) and specific geriatric syndromes in older adults with the Rapid Geriatric Assessment (RGA) tool. The RGA, which includes four geriatric syndromes: frailty, sarcopenia, geriatric anorexia, and cognitive decline, was administered to a total of 16,615 individuals aged 65 years and over across Missouri from 2015 to 2024. Nearly 40% of the participants (37.3%) reported having five or more illnesses. After controlling for demographic characteristics, logistic regression analysis showed that individuals with sarcopenia were over three times more likely to have multimorbidity compared to those without sarcopenia (OR = 3.807; CI: 3.488–4.156, *p* < 0.001). Similarly, the presence of geriatric anorexia and dementia was significantly associated with a 33% (OR = 1.329; CI: 1.224–1.443, *p* < 0.001) and 27% (OR = 1.273; CI: 1.158–1.401, *p* < 0.001) higher probability of having multimorbidity, respectively. This cross-sectional study provides evidence that the RGA is a valid screening tool for identifying individuals with multimorbidity across different practice settings. The findings underscore the importance of early detection of geriatric syndromes to prevent further morbidity and disability among older adult populations.

## Introduction

Many older adults (65+) in the United States (U. S.) often experience multimorbidity, defined as the coexistence of two or more chronic diseases. The prevalence of multimorbidity significantly increased from 45.7% in 1988 to 59.6% in 2013–2014 (1). Recent data show that this prevalence among adults aged 50 and older is 63.8 and 91.8% for adults aged 65 and older in the U. S. (2). Systematic reviews report that the prevalence of multimorbidity among older adults varies significantly across studies, ranging from 4.8 to 93.1%, depending on study design, population, and operational definitions used to assess multimorbidity (3, 4). Despite these variations, the overall trend of increasing multimorbidity associated with aging indicates the extensive impact and burden of multimorbidity with significant implications for healthcare needs (1). Older adults with multimorbidity are at heightened risk for acute illnesses, which can lead to further deterioration of their health and exacerbate existing conditions (5, 6). They also experience frequent hospitalizations and medical visits, leading to increased healthcare utilization and costs.

The increased prevalence of multimorbidity among older adults is a growing concern globally. Using 126 peer-reviewed studies including 15.4 million individuals across 54 countries, a recent systemic review and meta-analysis reported an overall prevalence of multimorbidity of 37.2% with regional estimates ranging from 35% in Asia to 45.7% in South America (7). More than half of aging population had multimorbidity (51.0%). Studies from specific countries (e.g., India, Hong-Kong, Portugal, Brazil) also highlighted the rising prevalence of multimorbidity among older adults (8–12). However, a number of cross-sectional studies have found that multimorbidity affects not only older adults but also a significant proportion of younger adults. For example, recent cohort studies in Spain, China, and the UK showed that younger adults, aged 18–65, experienced significant multimorbidity, with distinct patterns emerging early in adulthood (13, 14).

The overall aims of the United Nations Sustainable Development Goals (SDGs) are to achieve global health by providing a framework through multi-sectoral collaborations. Particularly, SDG 3 highlights the importance of ensuring healthy lives and promoting well-being for all, at all ages (15, 16), which aligns with addressing the challenges of multimorbidity in aging populations. Identifying the risk of multimorbidity at early life stage and its effective management directly contributes to promoting healthy aging by addressing the complex health needs of older adults. Thus, addressing the risk of multimorbidity through risk assessment supports global health priorities and the development of sustainable healthcare systems in geriatric care (16, 17).

Certain risk factors can increase the prevalence of multimorbidity among older adults. Age is consistently identified as one of the primary risk factors, with older populations experiencing a significantly higher incidence of multimorbidity. As individuals age, the likelihood of developing multiple chronic conditions rises sharply, a trend observed in numerous studies (18, 19). Socioeconomic status further contributes to the prevalence of multimorbidity. Research has shown that individuals living in areas of deprivation or those with lower household incomes or low-skilled occupations are at a higher risk of developing multiple chronic conditions (18–22). These individuals often experience barriers to accessing healthcare systems and resources or information for health-promoting, which may limit their ability to manage chronic conditions effectively.

Previous research demonstrated a significant overlap between geriatric syndromes and multimorbidity in older adults, with their prevalence rising steadily with age (23). These overlapping conditions are linked to increased healthcare utilization, such as more frequent hospital admissions and longer hospital stays, as well as adverse health outcomes, including functional decline, reduced quality of life, and heightened mortality risks (23, 24).

Comorbidity indices, such as the Charlson Comorbidity Index and Elixhauser Comorbidity Index (25–28), are widely used to quantify the burden of multiple chronic conditions. These indices have been associated with various geriatric syndromes, including frailty, polypharmacy, and functional decline, which are critical markers of vulnerability in older adults (29). Despite their utility, these indices may not fully capture the multi-faceted nature of geriatric syndromes, which often involve a broader range of factors like cognitive impairment, mobility limitations, and certain social determinants of health. This gap highlights the need for more comprehensive assessment tools tailored to geriatric populations, tools that can better account for the complexity of multimorbidity and its interaction with geriatric syndromes (29, 30).

Multimorbidity, encompassing both chronic diseases and geriatric syndromes, has been identified as a strong predictor of discharge challenges in older patients admitted to acute care hospitals. Older adults with multiple chronic conditions often face difficulties in transitioning from hospital care to home or other care settings, which complicates recovery and increases the likelihood of readmission (31). This challenge further emphasizes the need for holistic and integrated care approaches that consider the full scope of multimorbidity and geriatric syndromes to improve overall outcomes in older adults. Additionally, implementing a brief screening tool in regular wellness visits for older adults would help fill this gap. As such, this project utilized geriatric syndromes from the Rapid Geriatric Assessment (RGA) tool to assess its association with multimorbidity.

## Methods

### Rapid geriatric assessment

The Rapid Geriatric Assessment (RGA) was developed through initiatives on the Geriatric Workforce Enhancement Project (GWEP) as a brief screening tool to identify geriatric syndromes with four areas (frailty, sarcopenia, geriatric anorexia, and cognitive decline) (32–36). The RGA is comprised of the four separate, validated tools that collectively assess these syndromes (33, 35, 37–39). Administration takes approximately 5 min to complete all four measures. Due to its fair use, convenience, and user-friendly features, the RGA has been implemented to assess geriatric syndrome for over 18,000 individuals in various healthcare settings over the last 10 years.

### Sample and settings

We administered RGA screenings to individuals from July 2015 to June 2024. The RGA screening was conducted at 19 sites across a Midwestern state in the U.S. The sites include five types of locations: (1) acute care *hospitals* such as urban hospitals and emergency departments, a Veterans Administration Medical Center, and two rural critical access hospitals, (2) *physicians’ offices* in rural, suburban, and urban areas, (3) *screening* events occurring through community-based aging service organizations or agencies, health fairs, and educational events, (4) *post-acute and long-term care settings,* including skilled care facilities, and (5) *community programs*, such as a Program for All-Inclusive Care for the Elderly (PACE) (36, 40). More than 18,000 individuals were screened over the last 10 years and a total of 16,615 individuals who were 65 years and older were included in this study. Although the screenings were conducted over a 10-year period, no identifiers were collected. As a result, we were unable to track participants over time. Given this limitation, this study is based on a cross-sectional design, and any potential repeated assessments were treated as independent observations.

### Measures

Measures used in this study include participant’s personal characteristics and four geriatric syndrome measures from the RGA.

#### Personal characteristics

Participant’s variables included age, sex, race, and ethnicity. Age was categorized into three groups: 65–74, 75–84, and 85+. For sex, females were scored as 1. Race includes three categories: White/Caucasian, Black/African American, and Other. Ethnicity was asked whether a participant was Hispanic/Latino (=1) or non-Hispanic/Latino (=0).

#### Multimorbidity

To assess frailty, the RGA includes five questions on fatigue (i.e., whether you are fatigued), resistance (i.e., whether you can walk up one flight of stairs), aerobic capacity (i.e., whether you can walk one block), illnesses (i.e., whether you have more than five illnesses), and loss of weight (i.e., whether you have lost more than 5% of your weight in the last 6 months). In this study, multimorbidity was defined as having more than 5 illnesses using one question on illness from the frailty (33, 35).

#### Sarcopenia

Sarcopenia was assessed using five items on the loss of muscle: muscle strength, assistance in walking, rising from a chair, climbing a flight of stairs, and overall falls within the past year. Participants were asked about the level of difficulty when they performed four tasks, as well as the number of falls they had. Responses were scored on a scale of 0 (None), 1 (Some; 1–3 falls), and 2 (A lot or unable; 4 or more falls). The total score ranged from 0 to 10, with a total score of 4 or higher indicating sarcopenia (33, 37).

#### Geriatric anorexia

Geriatric anorexia was assessed using four simplified nutritional assessment questions. These questions asked about participant’s appetite, food tastes, feeling full when they eat, and the frequency of daily meals. Responses were scored on a scale from 1 to 5, where “1” indicated the poorest condition (e.g., very poor appetite, very bad food tastes, feeling full after only a few mouthfuls or eating fewer than one meal per day), and “5” indicated the best condition (e.g., very good appetite, very good food tastes, hardly ever feeling full, or eating more than three meals per day). The total score ranged from 4 to 20, with a score of 14 or below indicating a significant risk of at least 5% weight loss within 6 months (33, 39).

#### Cognitive decline

Cognitive decline was assessed using three items to screen for cognition and recall. Participants were asked to recall five objects (e.g., apple, pen, tie, house, car) after answering the subsequent question on a clock drawing. One point was scored for remembering each object correctly. The clock-drawing task was scored out of four points, with two points each for correctly indicating the time and hour. An additional point was given for correctly answering a story-based memory question. The total score ranged from 0 to 10, with scores of “8” or higher indicating normal cognitive function, “6 to 7” indicating mild cognitive impairment, and “5” or below indicating dementia (33, 38).

### Statistical analysis

We conducted descriptive statistics to analyze participant’s demographic characteristics and RGA measures. Specifically, we reported means and standard deviations for continuous variables and percentages within each category for nominal variable in the final analytic sample. T-tests and Chi-square tests were used to compare variability in RGA measures. A blockwise logistic regression model was performed to examine the factors associated with having 5 + illnesses. In the first model, participant’s characteristics (i.e., age group, sex, race, ethnicity) were included. Screening locations were added to Model 2. In Model 3, three measures from the RGA (i.e., sarcopenia, geriatric anorexia, and cognitive decline) were added. All analyses were done using IBM SPSS Statistics version 29.0. An IRB exemption was obtained for the RGA screening data, which was collected as anonymous data.

## Results

### Participant characteristics

Table 1 presents participant’s characteristics overall and by multimorbidity status. Overall, participants aged from 65 to 74 years old were 38.5, 36.1% for 75 to 84 years, and 25.4% for 85 years and older. A significantly higher proportion of participants aged over 85 years and older (28.8%) was observed in the group of having 5 + illnesses (χ^2^ = 118.08, *p* < 0.001) compared to the group of having fewer than 5 illnesses (23.4%). Most participants were female (64.4%) and White/Caucasian (75.9%), while the group of having 5 + illnesses had a slightly higher proportion of female (65.5%) (χ^2^ = 5.78, *p* < 0.05) and Black/African American participants (20.9%) (χ^2^ = 8.61, *p* < 0.05). Less than 1% were Hispanic. The screening location for the RGA was significantly associated with multimorbidity status (χ^2^ = 920.85, *p* < 0.001). The RGA was screened in acute care settings the most (44.3%) and followed by outpatient clinics (27.1%) in the group having 5 + illnesses while outpatient clinics (41.7%) were the most frequent location followed by acute care settings (36.4%) in the group having fewer than 5 illnesses. All four measures of the RGA were significantly different by multimorbidity status. Participants having 5 + illnesses had higher scores in frailty (*t* = 96.18, *p < 0*.001), sarcopenia (*t* = 46.59, *p < 0*.001), geriatric anorexia (*t* = 21.24, *p < 0*.001), and cognitive impairment status (*t* = 17.47, *p < 0*.001) than those having fewer than 5 illnesses.

**Table 1:** Comparison of participant characteristics by multimorbidity status (5 + Illnesses vs. Fewer than 5 Illnesses).

Characteristics	Having 5 + illnesses *N* (%)	Not having 5 + illnesses *N* (%)	Total *N* (%)
Age group^***^			
65–74	1,931 (33.2%)	4,074 (41.6%)	6,005 (38.5%)
75–84	2,211 (38.0%)	3,426 (35.0%)	5,637 (36.1%)
85+	1,678 (28.8%)	2,296 (23.4%)	3,974 (25.4%)
Sex^*^			
Male	1,993 (34.4%)	3,530 (36.3%)	5,523 (35.6%)
Female	3,802 (65.6%)	6,194 (63.7%)	9,996 (64.4%)
Race^*^			
White/Caucasian	4,340 (74.6%)	7,508 (76.6%)	11,848 (75.9%)
Black/African American	1,218 (20.9%)	1,877 (19.2%)	3,095 (19.8%)
Other or not reported	262 (4.5%)	411 (4.2%)	673 (4.3%)
Hispanic	43 (0.8%)	87 (0.9%)	130 (0.9%)
Screening location^***^			
Acute care settings	2,580 (44.3%)	3,570 (36.4%)	6,150 (39.4%)
Nursing homes	675 (11.6%)	184 (1.9%)	859 (5.5%)
Outpatient clinics	1,578 (27.1%)	4,084 (41.7%)	5,662 (36.3%)
Screening	902 (15.5%)	1,832 (18.7%)	2,734 (17.5%)
PACE	85 (1.5%)	126 (1.3%)	211 (5.5%)
Frailty^***^	2.71 (±1.19)	0.91 (±1.09)	1.58 (±1.42)
Rapid Cognitive Screen (RCS)^***^	6.45 (±3.08)	7.35 (±2.67)	6.98 (±2.89)
SARC-F for Sarcopenia^***^	4.40 (±3.09)	2.20 (±2.65)	3.06 (±3.03)
Simplified Nutritional Appetite Questionnaire (SNAQ)^***^	14.13 (±2.69)	15.04 (±2.44)	14.69 (±2.57)

*N* = 16,615. Total N varies due to missing cases; ^*^p < 0.05, ^***^p < 0.001; PACE: Programs of All-Inclusive Care for the Elderly.

### Correlates of multimorbidity status

Table 2 shows the results from the blockwise logistic regression. The mid-old age group (75–84 years) was significantly more likely to report having 5 + illnesses (OR = 1.200, 95% CI = 1.090–1.320, *p* < 0.001), whereas the old-old age group (85 + years) was less likely to do so (OR = 0.892, 95% CI = 0.798–0.997, *p* = 0.044). Female participants were less likely to report having 5 + illnesses compared to male participants (OR = 0.913, 95% CI = 0.838–0.995, *p* = 0.037). Black/African American participants were significantly more likely to report having 5 + illnesses compared to White/Caucasians (OR = 1.270, 95% CI = 1.145–1.409, *p* < 0.001). The odds of having 5 + illnesses were also higher for screenings administered at nursing homes (OR = 3.040, 95% CI = 2.498–3.699, *p* < 0.001) and lower at physician offices (OR = 0.545, 95% CI = 0.498–0.598, *p* < 0.001), community screening (OR = 0.505, 95% CI = 0.446–0.571, *p* < 0.001), and PACE (OR = 0.670, 95% CI = 0.458–0.979, *p* = 0.039), compared to being screened in acute care settings.

**Table 2:** Logistic regression predicting having 5 + illnesses.

	Having 5 + illness	*p*
	OR (95% CI)	
Age group		
65–74 (reference group)	–	
75–84	1.200 (1.090–1.320)	< 0.001
85+	0.892 (0.798–0.997)	0.044
Female (vs. male)	0.913 (0.838–0.995)	0.037
Race		
White/Caucasian (reference group)	–	
Black/African American	1.270 (1.145–1.409)	<0.001
Other or not reported	0.956 (0.750–1.219)	0.716
Hispanic (vs. no Hispanic)	0.766 (0.493–1.190)	0.236
Screening location		
Acute care settings	–	
Nursing homes	3.040 (2.498–3.699)	<0.001
Outpatient clinics	0.545 (0.498–0.598)	<0.001
Screening	0.505 (0.446–0.571)	<0.001
Programs of All-Inclusive Care for the Elderly (PACE)	0.670 (0.458–0.979)	0.039
Rapid cognitive screen (RCS)		
Normal (reference group)	–	
Mild cognitive impairment (MCI)	1.113 (1.000–1.237)	0.049
Dementia	1.128 (1.022–1.245)	0.017
SARC-F for sarcopenia		
No sarcopenia (reference group)		
Sarcopenia	3.480 (3.180–3.807)	<0.001
Simplified nutritional appetite questionnaire (SNAQ)		
No risk of weight loss (reference group)	–	
Risk of weight loss	1.355 (1.246–1.474)	<0.001

*N* = 12,035.

Cognitive status, sarcopenia, and geriatric anorexia were also significantly associated with the odds of having 5 + illnesses. Participants having sarcopenia were more than three times as likely to report having 5 + illnesses (OR = 3.480, 95% CI = 3.180–3.807, *p* < 0.001) than those without sarcopenia. Compared to participants with normal cognitive status, those having mild cognitive impairment (MCI) (OR = 1.113, 95% CI = 1.000–1.237, *p* = 0.049) and dementia (OR = 1.128, 95% CI = 1.022–1.245, *p* = 0.017) were significantly more likely to report having 5 + illnesses. Lastly, participants who were at risk of weight loss were more likely to report having 5 + illnesses (OR = 1.355, 95% CI = 1.246–1.474, *p* < 0.001) than those without the risk of weight loss.

## Discussion

Around half of the older adult population in the U. S. struggles with managing multiple chronic conditions, having two or more chronic illnesses simultaneously. As previously introduced, the impact of multimorbidity is significant, including increased vulnerability to sudden illnesses, worsening of pre-existing conditions, frequent hospital stays, and high healthcare costs (5, 6). Early identification of the risk factors associated with multimorbidity can help healthcare planning and potentially prevent adverse health outcomes (19). Using a brief geriatric syndrome assessment across different practice settings in a Midwestern state, this study provides evidence that the RGA is a valid screening tool that is associated with the presence of multimorbidity in older adults. The higher odds of having 5 + illnesses were significantly associated with the prevalence of sarcopenia, geriatric anorexia, and neurocognitive disorders after adjusting demographic characteristics.

This study is unique in using a screening tool administered over 10 years to detect geriatric issues. The RGA was developed in 2015 to identify treatable geriatric syndromes, which would help clinicians’ early detection of certain conditions and help improve patient-centered care (35, 36). We found significant associations among the prevalence of sarcopenia, geriatric anorexia, neurocognitive disorders, and having five or more illnesses, which extend previous findings from data examining the RGA from 2015 to 2019 (36). The results emphasize the importance of routinely identifying geriatric syndromes in clinical practice and highlight the benefit of the RGA not only as an effective brief screener but also a significant predictor of potential geriatric conditions that need more in-depth follow-up in geriatric patient care.

A key takeaway from this study is that we identified demographic characteristics associated with a higher likelihood of reporting multimorbidity. Participants in the mid-old age group (74–84 years), males, Black/African Americans, and those residing in nursing homes had significantly greater odds of having 5 + illnesses compared to other groups. In other words, compared to the groups aged 65 to 74, the mid-old group (74–84 years) showed 20% more likely to report having multiple chronic conditions, whereas the oldest-old group (85 + years) had 10.8% lower odds. Further, females had 8.7% lower odds of reporting multimorbidity than males. The survivor effect may explain these significant findings: women and those who aged 85 years and older might have been in better physical condition than men and those who died earlier, respectively (41). Moreover, cumulative disadvantage related to healthcare access resulting from social and structural forces may explain why Black/African Americans showed 27% higher odds of having multimorbidity than White/Caucasians. Highlighting the need to improve access to geriatric care for vulnerable patient populations and reduce disparities in providing better care, these findings call for targeted community-based programs, culturally tailored interventions and expanded geriatric services in high-risk settings. Early and ongoing assessment of common geriatric syndromes has important implications for both practice interventions and policy development. While identifying the presence of the four common geriatric syndromes (i.e., sarcopenia, geriatric anorexia, frailty, and cognitive decline) included in the RGA does not ensure reversal, cure, or delayed disease progression, early detection would provide opportunities for ethical and effective responses. For those syndromes that will continue to progress (e.g., dementia), early assessment would enable the person experiencing cognitive impairment to be actively engaged in planning for their future. As a secondary preventive strategy, the RGA can enable older adults, their caregivers, and the healthcare team to implement pharmacologic to delay disease progression and non-pharmacologic interventions to modify lifestyle and improve quality of life.

Lastly, the findings underscore the value of early diagnosis and treatment of age-related issues in geriatric patient care across diverse practical settings. The RGA is an effective and efficient tool not only for screening common geriatric syndromes, which often go overlooked or under-assessed, but also for identifying and developing care plans for older adults experiencing multimorbidity. Because administering the RGA does not require clinical training, incorporating it into routine appointments with older patients can be effective, given the short period of time needed to administer the screening. Furthermore, this brief screening tool can be easily integrated into practice and electronic healthcare records (EHR), which can also strengthen the interprofessional healthcare team, as the tool can be completed and shared with the various disciplines comprising the interprofessional team for monitoring older patients’ risk factors or indicators of higher healthcare use. As the geriatric syndromes included in the RGA are often under-assessed, thus missing opportunities for treatment or intervention, it is critical for providers of all disciplines to be aware of and trained to competently administer such screening tools, particularly in light of the findings of this study related to identification of multimorbidity. Moreover, utilizing information embedded in EHR would serve as a basis for active geriatric care and contribute to the recognition of an age-friendly healthcare system. Early assessment and intervention can have significant implications for healthcare costs and system-level planning. Preventing or managing chronic conditions in their early stages has the potential cost-savings when compared to treatment at a later stage. For example, medical annual wellness visits have been associated with a 5.7% reduction in total healthcare costs (42), and primary care-adapted comprehensive geriatric assessment is cost-effective (43). In alignment with the goals of the Medicare Annual Wellness Visit, healthcare organizations and insurers could adopt the RGA as a standardized protocol to identify at-risk older adults, guide preventive care, and inform equitable resource allocation.

While the RGA is a useful and convenient tool for assessing multimorbidity in geriatric patients, some limitations should be considered. First, we assessed multimorbidity based on a single self-reported item from the RGA, asking whether participants had more than five illnesses. Incorporating clinical data such as medical diagnoses (e.g., ICD-10 codes) would provide a comprehensive estimation of disease burden by enhancing the accuracy and validity of the multimorbidity assessment. Second, the RGA screening tool does not cover all risk factors practitioners should be mindful of when evaluating geriatric patients. Including other assessment measures to examine behavioral risk factors (e.g., physical activity, smoking) and mental health (e.g., depression, anxiety) would provide a more comprehensive understanding of a patient’s complex needs. While the RGA can provide considerable information related to multimorbidity and risk factors, it is a screening tool rather than a diagnostic tool and should be interpreted accordingly. Furthermore, the dataset collected over a 10-year period did not include participant identifiers for participants, which prevented us from tracking individuals over time. A longitudinal design would allow for follow-up assessments to examine the progress of geriatric syndromes and the development of multimorbidity. Moreover, future longitudinal studies would contribute to establishing causal directions between geriatric syndromes and multimorbidity. Additional limitations include that the data was collected from one geographic area, Missouri, which is overwhelmingly composed of White participants. While older Black/African American adults represent nearly a fifth of this sample, it lacks representation from other races and ethnicities. Environmental factors play a significant role in one’s health (44, 45), and Missouri is primarily a rural, Midwestern state with four larger urban cities (St. Louis, Kansas City, Springfield, and Columbia). This study did not include patient’s educational level or household income, which are important determinants of multimorbidity. A recent cross-sectional study across 33 countries found that individuals with lower levels of education and income had higher odds of experiencing multimorbidity (46). To address these social inequalities in multimorbidity, future research should incorporate education and income levels, which will provide evidence that can inform health policies. Such evidence would support implementing resource redistribution and targeting health resources and interventions for less advantaged patient populations. Finally, the nursing home sample is very small, which suggests findings for this population are not generalizable. Future research is needed to understand multimorbidity among this segment of the aging population.

In addition to conducting further research with geriatric patients in post-acute and long-term care settings, which was a relatively underrepresented group in this study, more examination should be conducted about the return on investment for organizations or practitioners using valid, efficient geriatric assessment tools. For example, studies should examine whether the early identification and treatment of common geriatric syndromes reduce hospitalizations, length of stay and placement, and healthcare costs. There is also a need to establish best practices for transforming current geriatric assessment methods into more evidence-based, age-friendly strategies. Policies and resources at the national level are needed to support age-related assessments and the management of multimorbidity. As an example, the new CMS Final Rule mandates that acute care organizations incorporate the Age-Friendly Health Systems (AFHS) 4Ms framework into the workflow for inpatient care, representing a step in the right direction. The RGA could be a valuable tool adopted throughout systems to promote consistency in measuring older adults’ physical and cognitive health.

## Conclusion

Overall, the RGA is a valid and efficient screening tool for identifying individuals with high levels of multimorbidity across different practice settings. Future research should explore whether it can also identify those at risk of developing multimorbidity. Older adults with sarcopenia, geriatric anorexia, and cognitive decline were significantly more likely to have five or more chronic conditions. Integrating the RGA into routine care may help identify older adults with high levels of multimorbidity and inform care planning, which ultimately contributes to global health priorities by promoting healthy aging. With the growing number of older adults experiencing increased chronic and life-threatening conditions, detecting these health issues earlier for this population is critical in the coming years.

## Data Availability

The original contributions presented in the study are included in the article/supplementary material, further inquiries can be directed to the corresponding author/s.
